# Level of evidence in hand surgery

**DOI:** 10.1186/1756-0500-5-665

**Published:** 2012-12-02

**Authors:** Roberto S Rosales, Luis Reboso-Morales, Yolanda Martin-Hidalgo, Isabel Diez de la Lastra-Bosch

**Affiliations:** 1 Unit for Hand & Microsurgery, GECOT, Maria del Cristo Ossuna 20, La Laguna, Tenerife, 38204, Spain; 2 Orthopaedic Surgery Service, University Hospital of “La Candelaria”, Carretera del Rosario s/n, La Laguna, Tenerife, 38110, Spain; 3 Government Health Service, Oncology Programme, Canarian Health Service, Pérez de Rozas 5, 2nd floor, Santa Cruz de Tenerife, Tenerife, 38005, Spain

**Keywords:** Hand, Level of evidence, Hand surgery, Clinical research

## Abstract

**Background:**

Few investigations have been done to analyze the level of evidence in journals related to hand surgery, compared to other related research fields. The objective of this study was to assess the level of evidence of the clinical research papers published in the Ibero-american (RICMA), the European (JHSE) and American (JHSA) Journals of Hand Surgery.

**Methods:**

A total of 932 clinical research papers published between 2005 and 2009 (RICMA 60, JHSE 461, and JHSA 411) were reviewed. Two independent observers classified the level of evidence based on the Oxford International Classification, 5 being the lowest level and 1 the highest level. The observed frequencies of the level of evidence for each journal were compared with the expected frequencies by a chi-square (χ ^2^) test for categorical variables with a significance level of 0.05.

**Results:**

Inter-observer agreement analysis showed a Kappa of 0.617. Intra-observer agreement analysis presented a Kappa of 0.66 for the observer 1, and a Kappa of 0.751 for the observer 2. More than 80% of the papers in RICMA and JHSE and a 67.6% in the JHSA presented a level of 4. No level 1 or 2 studies were published in RICMA, compared to JHSE (0.9% level 1 and 5.0% level 2) and JHSA (8.3% level 1 and 10% level 2). The percentage of papers with level 3 published in RICMA (16.7%) was higher compared to the JHSE (11.1%) and the JHSA (14.1%). All the results were statistically significant (χ^2^=63.945; p<0.001).

**Conclusions:**

The level of evidence in hand surgery is dependent on the type of journal; being the highest level evidence papers those published in the JHSA, followed by the JHSE and finally the RICMA. Knowing the status of the level of evidence published in hand surgery is the starting point to face the challenges of improving the quality of our clinical research

## Background

Since the first system for classifying the level of evidence of the clinical research papers was reported [1], Evidence-Based Medicine has become an important part of our clinical practice. Hand surgeons should understand the level of evidence in order to become aware of the reliability and the utility of the data provided in a research paper. Few investigations have been done to analyze the level of evidence in journals related to hand surgery compared to other related research fields, as in the orthopaedic surgery [2,3], and plastic surgery [4] journals. Only one specific hand surgery journal has been analyzed for evidence level over a six month period of time, and has been compared to other orthopaedic publications [3]. To our knowledge, no paper comparing the level of evidence in clinical research published in three hand surgery specific journals over a period of five years, has been reported before. The purpose of this paper was to assess the level of evidence of the clinical research papers published between 2005 and 2009 in the Ibero-American Journal of Hand Surgery (RICMA), as the official journal of the Spanish, Portuguese and the main Latin American Societies for Surgery of the Hand; the European (JHSE) and American (JHSA) Journals of Hand Surgery, as the official journals of the Federation of European Societies for Surgery of the Hand (FESSH) and the American Society for Surgery of the Hand (ASSH).

### Hypothesis

The researchers established the null hypothesis (Ho) that the variable “level of scientific evidence” was independent of the variable “type of journal”.

## Methods

### Eligibility criteria and population study

Inclusion criteria. All the clinical research articles, which were published between January 2005 and December 2009 in the: Ibero-American Journal of Hand Surgery (RICMA) (“Revista Iberoamericana de Cirugía de la Mano”); The Journal of Hand Surgery European Volume (JHSE) and The Journal of Hand Surgery American Volume (JHSA).

Exclusion criteria. Animal studies, anatomical and cadaver studies, basic science studies, instructional course lectures, supplements of abstract, short reports, letters to the editors and review articles were not considered as feasible for the study.

Hence, a total of 932 clinical research papers followed the inclusion and exclusion criteria (RICMA 60, JHSE 461, and JHSA 411).

### Assessment of level of evidence

The articles which met the inclusion and exclusion criteria were randomly assigned to two independent observers (YMH, LRM), with large clinical experience in hand surgery, and very familiar with Evidence-Based Medicine. An approximate equal number of articles from each journal were assessed by each observer. The level of evidence for each article was assessed based on The Oxford Centre for Evidence-based Medicine - Levels of Evidence (March 2009) http://www.cebm.net/index.aspx?o=1025[5]. The observers were blinded to any previous information related to the level of evidence of the articles to be analysed (e.g.: “level of evidence and type of study”, which is included in The JHSA with the abstract of every clinical research paper since 2006). The articles were ranked according to their level of evidence from Level 1 (highest level of evidence, e.g.: systematic review (SR), meta-analysis (MA), and individual randomized controlled trials (RCT) with narrow interval confidence) to level 5 (lowest level of evidence, e.g.: expert opinion). Assessment of the sub-group level of evidence, as well as the type of research article was not done by the observers (Table 1).

**Table 1:** Level of evidence and type of study

	**Therapy /Prevention, Aetiology/Harm**	**Prognosis**	**Diagnosis**	**Differential diagnosis/ symptom prevalence**	**Economic and decision analysis**
		**Investigating the effect of patient characteristic on the outcome of disease**	**Investigating a diagnostic test. Is this diagnostic test accurate?**		
**Level 1**	Systematic Review of randomized trials(RT)	Systematic Review of inception cohort studies	Systematic Review of level 1 diagnostic studies	Systematic Review of prospective or classic cohort	Systematic Review of level 1 economic studies
	High quality **RT**(e.g.:> 80% follow up, narrow confident interval)	Individual **cohort** study with > 80% follow up, all patient enrolled at the same time	Level 1 diagnostic studies or **Validating** studies which test the quality of a specific diagnostic test, previously developed, in series of consecutive patients with reference “gold” standard	Prospective or **classic cohort** studies with good follow up (>80%)	Level 1 studies (analysis based on clinically sensible costs or alternative, values obtained from **many studies**, and including multiway sensitive analysis
**Level 2**	Systematic Review of cohort studies	Systematic Review of either historical cohort study or untreated control groups (control arm) in RCTs	Systematic Review of level 2 diagnostic studies	Systematic Review of level 2 studies	Systematic Review of level 2 studies
	Lesser quality **RT **(e.g.: <80% follow up, wide confident interval, no clear randomization, problems with blinding, etc.)	**Historical** (retrospective) **cohort** study or control arm from a RCT	Level 2 diagnostic studies or **Exploratory** studies which collect information, trawl data to find which factor are significant (e.g.: using regression analysis)	Level 2 studies (retrospective or **historical cohort** study or with follow up <80%)	Level 2 studies (analysis based on clinically sensible cost or alternative from **limited** studies, and including multiway sensitivity analysis.
	Individual **Cohort** study, including matched cohort studies (prospective comparative studies)			Ecological Studies	
	Ecological Studies				
**Level 3**	Systematic Review of case–control studies		Systematic Review of level 3 studies	Systematic Review of level 3 studies	Systematic Review of level 3 studies
	Individual **case**–**control** study		Level 3 diagnostic studies or studies in non-consecutive patients and **without** consistently reference “gold” standards	Level 3 studies (**non**-**consecutive** cohort or very limited population)	Level 3 studies (analysis based on poor alternative or costs, **poor quality** estimates of data, but including sensitivity analysis
**Level 4**	Case-series	Case-series	Case–control study	Case-series	No sensitivity analysis
	Poor quality cohort and case–control studies*	Poor quality cohort and case–control studies*	Poor or non independent reference standard		
**Level 5**	Expert opinion	Expert opinion	Expert opinion	Expert opinion	Expert opinion

A systematic review (SR) is generally better than an individual study. Experimental study (e.g.: good quality RCT) is generally better than any observational study. For observational studies : cohort study is generally better than any case–control study . A case- control study is generally better than any case- series study. * By poor quality cohort study we mean a cohort study that failed to clearly define comparison groups and/or failed to measure exposures and outcomes (preferable blinding) in the same objective way in both expose and non-exposed individuals and/or failed to identify control known confounders and/ or poor follow up. The same for poor quality case–control study except that the patients are identified based on the outcomes in this design ( e.g.: failed replant) called “cases” are compared with those who did not have the outcome (e.g.: had a successful replant) called “controls” and consequently we do not have “exposed and non-exposed” and “longitudinal follow up”. Ecological studies and Economic/decision analysis studies are very uncommon in hand surgery. *This chart was adapted from material published by the Centre for Evidence-Based medicine, Oxford, Uk. March 2009*.

### Reliability analysis

Before starting the study, the reliability of the assessment was evaluated based on the analysis of both the intra-observer error and inter-observer error. A random sample of 30 clinical research articles, from a total of 872 papers, published in the English language (461 from JHSE, and 411 from JHSA), were assessed by the two independent observers assigned to the study. After 15 days, a second assessment was undertaken with the order of the articles changed. No papers from the RICMA were included in the sample study for the reliability analysis. This was done so as to avoid information bias [6], because the different languages present in the RICMA publication (Spanish and Portuguese), could increase the intra-observer reliability. The intra-observer and inter-observer reliability was studied using the Kappa coefficient test with a significance level of 0.05.

### Data analysis

For the assessment of the results, the number of articles for each level of evidence rating was expressed as a percentage of the total number of articles meeting the inclusion and exclusion criteria for the period time study. The observed frequencies of the level of evidence for each journal were compared with the expected frequencies using a chi-square (χ ^2^) test for categorical variables with a significance level of 0.05.

## Results

Inter-observer analysis showed a Kappa of 0.617, with an asymptotic standard error of Kappa (SE) of 0.117. Intra-observer analysis presented a Kappa of 0.66 (SE 0.114) for observer 1, and a Kappa of 0.751 (SE 0.103) for observer 2. All Kappa values were significant (p< 0.001). More than 80% of the papers in the RICMA and the JHSE; and a 67.6% in the JHSA presented a level of 4. Not one of the level 1 and 2 papers was published in the RICMA compared to the JHSE (0.9% level 1 and 5.0% level 2) and the JHSA (8.3% level 1 and 10% level 2). The percentage of papers with level 3, published in the RICMA (16.7%), was higher compared to the JHSE (11.1%) and the JHSA (14.1%) (Table 2). All the results were statistically significant (χ^2^=63.945; p<0.001) and the null Hypothesis (Ho) was rejected.

**Table 2:** **Crosstabulation of **“**type of journal**” **and** “**level of evidence**”

**Journal**	**Level of evidence**					**Total number of papers (n**)
	**1**	**2**	**3**	**4**	**5**	
RICMA	0.0%	0.0%	16.7%	80.0%	3.3%	
(CI 95%)	(N.A)	(N.A)	(7.3 ; 26.1)	(69.9 ; 90.1)	(N.A.)	
	0	0	10	48	2	60
JHSE	0.9%	5.0%	11.1%	82.4%	0.7%	
(CI 95%)	(N.A.)	(3.02 ; 6.9)	(8.3 ; 13.9)	(79 ; 85.8)	(N.A.)	
	4	23	51	380	3	461
JHSA	8.3%	10%	14.1%	67.6%	0%	
(CI 95%)	(5.6 ; 10.9)	(7.1 ; 12.9)	(10.7 ; 17.4)	(63.1 ; 72.1)	(N.A.)	
	34	41	58	278	0	411

RICMA = IBero-american Journal of Hand Surgery, JHSE= Journal of Hand Surgery European Volume, JHSA= Journal of Hand Surgery American Volume. (CI 95%) = 95% Confidence Interval. (N.A.) = CI 95% is not applicable when the observed proportion is not greater than 5/n.

## Discussion

Results of this paper have demonstrated with a good – excellent level of reliability that the variable “level of evidence” is dependent on the variable “type of journal”.

### Reliability analysis

The use of Kappa is important, as an often used proportion of agreement does not allow for the fact that some agreement is due to chance. A statistically significant Kappa coefficient means that the agreement is different from zero (null agreement). However, the interpretation of obtained values of kappa is subjective, and different classifications or guides have been proposed to interpret the Kappa coefficient in the reliability analysis. In this paper, the level of agreement in the inter observer and intra observer analysis has shown that a kappa value ranging from 0.617 to 0.751, can be considered as having an excellent to a good level of reliability [7,8] in the assessment of the level of evidence and the type of journal. Similar results have been reported before. Obremskey et al. [3], in the assessment of the level of evidence in orthopaedic journals, have reported Kappa values of 0.62 for inter observer agreement between inexperienced reviewers, and a kappa value of 0.75 for inter observer reliability between experienced reviewers. No intra observer agreement analysis was reported by those authors.

### Level of evidence and type of journal

Not many papers have studied the level of evidence in hand surgery journals or in related research fields, such as orthopaedic and plastic surgery journals. Sinno et al. [4], reviewed 726 from six different plastic surgery journals and the level of evidence was assessed using a classification based on the Oxford Centre for Evidence level (CEBM). Hanzlik et al. [2] assessed 551 papers from the Journal of Bone Joint Surgery American Volume (JBJSA) from the years 1975 (134 papers), 1985 (123 papers), 1995 (120 papers), and 2005(174 papers). The level of evidence was assessed using a classification included in the guide for authors (JBJS-A grading system) which was very similar to the one developed by the CEBM, in order to demonstrate trends in the level of evidence over 30 years. Furthermore, Obremskey et al. [3] reviewed 382 clinical research articles from nine different journals in order to assess the level of evidence in orthopaedic journals. In this paper, 932 clinical research papers from three specific hand surgery journals were reviewed, which constitutes the largest population of scientific clinical articles assessed to study the level of evidence reported until now.

The results of this paper demonstrate that most of the clinical articles published in hand surgery, are papers with a very low level of evidence (80% level 4 in the JHSE or RICMA and 67.6% in the JHSA). Most of those papers were case-series and less frequently, poor quality cohort or poor quality case–control studies. Those results were higher compared to orthopaedic journals (48 % level 4 studies) [2], to plastic surgery journals (40% level 4 studies) [4] and to ophthalmology journals (58% Level 4 studies) [9]. However, other surgical journals as ear, nose and throat (otolaryngology) journals present a percentage similar to JHSE and RICMA (80% Level 4 studies) [10]. The percentage of level 4 papers in JHSA was lower, as compared to the rest of the hand surgery journals investigated, and it was very close to the one published by Obremskey et al. [3], who reported a 68.8% of level 4 papers, in a review of 32 articles published in the JHSA from January to June 2003.

The percentage of papers with a higher level of evidence (level 1 and 2), was larger in the JHSA (8.3% level 1 and 10% level 2), compared to the RICMA (0%) and the JHSE (0.9% level 1 and 5% level 2). Whilst compared to other journals, there was 21% of level 1 and 15% of level 2 of evidence in orthopaedic journals [2], 3% of level 1 and 16% of level 2 in plastic surgery journals [4], 18% of level 1 and 8% level 2 in ophthalmology journals [9], and 7% of level 1 and level 2 in otolaryngology journals [10].

The percentage of papers with level 3 (mostly case- control studies and non-consecutive cohort studies or with very limited population) published in the RICMA (16.7%) was higher compared to the JHSE (11.1%) and the JHSA (14.1%); and similar to other journals: 16% in orthopaedic journals [2], 16% in otolaryngology journals [10] and 16% in ophthalmology journals [9]. Hence, some authors have criticized the low number of high evidence level in surgery [11]. Even so, the criticism may seem overly severe, if we take into account that surgical trials are different from trials, which compare a medication with a placebo. Surgical procedures are invasive; it is difficult to randomise patients, blinding is a problem in surgical trials, and they are very expensive. If we do not have high quality randomized trials we cannot have a systematic review which synthesizes the evidence previously reported.

No trend analysis is a limitation for this paper, and the information within should be the purpose of further studies, in order to understand how the evidence published in hand surgery journals has changed and how the relationship between changes in the level of evidence and changes in the impact factor index, have also changed over time.

After reviewing several articles published in journals from different parts of the world, other questions have arisen. These being, whether the differences that we have found are a reflection of different regional priorities or how the resources used for research have an impact on our findings and even if different countries are the main contributors in high level studies.

## Conclusions

The level of evidence in hand surgery is dependent on the type of journal; being the highest level evidence papers those published in the JHSA, followed by the JHSE and finally the RICMA. Knowing the status of the level of evidence published in hand surgery is the starting point to face the challenges of improving the quality of our clinical research.

## Competing interests

There are no financial or non-financial competing interests to declare in relation to this manuscript.

## Authors’ contributions

RSR was involved as director of the study. He devised and designed the study and was also involved in the analysis and the interpretation of the data. LRB & YMH were the two independent observers involved in the reliability analysis and in the assessment of the level of evidence of papers which followed the inclusion and exclusion criteria. IDLB was involved in the acquisition and analysis of the data, as well as in the drafting and reviewing of the manuscript. All the authors read and approved the final version of the manuscript.

## References

[B1] Sackett DL Rules of evidence and clinical recommendations on the use of antithrombotic agents Chest 1986 89 2 Suppl 2S 3S 3943408

[B2] Hanzlik S Mahabir RC Baynosa RC Khiabani KT Levels of evidence in research published in the journal of bone and joint surgery (American volume) over the last thirty years J Bone Joint Surg 2009 91 A 425 428 19181987 10.2106/JBJS.H.00108

[B3] Obremskey WT Pappas N Attallah-Wasif E Tornetta P Bhandari M Level of evidence in orthopedic journals J Bone Joint Surg 2005 87 A 2632 2638 16322612 10.2106/JBJS.E.00370

[B4] Sinno H Neel OF Lutfy J Bartlett G Gilardino M Level of evidence in plastic surgery research Plast Reconstr Surg 2011 127 974 980 10.1097/PRS.0b013e318200af74 21285804

[B5] Oxford Centre for Evidence-based Medicine Levels of Evidence Available at: http://www.cebm.net/index.aspx?o=1025. Accessed March 2009

[B6] Page RM Cole GE Timmreck TC Basic Epidemiological Methods and Biostatistics. A practical guidebook 1995 Boston: Jones and Bartlett publishers

[B7] Landis JR Koch GG The measurements of observer agreement for categorical data Biometrics 1977 33 159 174 10.2307/2529310 843571

[B8] Silman AJ Epidemiological Studies: a practical guide 1995 New York: Cambridge University Press

[B9] Lai TY Leung GM Wong VW Lam RF Cheng AC Lam DS How evidence-based are publications in clinical ophthalmic journals? Invest Ophthalmol Vis Sci 2006 47 1831 1838 10.1167/iovs.05-0915 16638988

[B10] Bentsianov BL Boruk M Rosendfield RM Evidence-based medicine in otolaryngology journals Otolaryngol Head Neck Surg 2002 126 371 376 10.1067/mhn.2002.123859 11997775

[B11] Horton R Surgical research or comic opera: questions, but few answers Lancet 1996 347 984 985 10.1016/S0140-6736(96)90137-3 8606606

